# Use of a portable monitoring device (Somnocheck Micro) for the investigation and diagnosis of obstructive sleep apnoea in comparison with polysomnography

**DOI:** 10.12669/pjms.322.9561

**Published:** 2016

**Authors:** Cahit Bilgin, Unal Erkorkmaz, Muhammed Kursad Ucar, Nese Akin, Ahmet Nalbant, Ali Nihat Annakkaya

**Affiliations:** 1 Dr. Cahit Bilgin, MD. Department of Chest Diseases, Sakarya University Medical School, Sakarya, Turkey; 2 Dr. Unal Erkorkmaz, PhD. Department of Biostatistics, Sakarya University Medical School, Sakarya, Turkey; 3 Eng. Muhammed Kursad Ucar, Department of Electrical Electronics Engineering, Sakarya University, Sakarya, Turkey; 4 Dr. Nese Akin, MD. Bursa Prof. Dr. Turkan Akyol Goverment Hospital of Chest Diseases, Bursa, Turkey; 5 Dr. Ahmet Nalbant, MD. Sakarya Training and Research Hospital of Internal Medicine, Sakarya, Turkey; 6 Prof. Ali Nihat Annakkaya, MD. Department of Chest Diseases, Duzce University Medical School, Duzce, Turkey

**Keywords:** Obstructive sleep apnoea syndrome, portable sleep device, polysomnography, photoplethysmography, Pulse wave analysis

## Abstract

**Objective:**

Polysomnography (PSG) remains the gold standard for the diagnosis of obstructive sleep apnoea syndrome (OSAS). While PSG is essential for OSAS, this technique is not suitable for epidemiological investigation due to its high cost. This study aimed to compare a portable monitoring device with PSG for the measurement of parameters related to the diagnosis of OSAS in rural areas.

**Methods:**

We conducted a descriptive study of 155 patients (30 women and 125 men; mean age, 52±12years) who visited to the Hendek Government Hospital Sleep Laboratory between February 2011 and January 2013 Apnoea hypopnea index (AHI), mean levels of O_2_ (meanO_2_), desaturation index (DI), and minimum oxygen saturation (minO_2_) variations as measured using both PSG and a portable Somnocheck Micro (SM) device were compared.

**Results:**

Differences were found between the meanO_2_ and DI, but not between AHI and minO_2_. Differences between the methods were not desired, but the relationship between the methods was distinct and supported our hypothesis.

**Conclusions:**

The results of our study have shown that the SM portable device can be used as an alternative diagnostic tool in this population either at home or in sleep clinic.

## INTRODUCTION

The current standard for clinical practice, established through evidence-based reviews by the American Academy of Sleep Medicine (AASM) is to confirm the diagnosis of Obstructive sleep apnoea syndrome (OSAS) with in-laboratory Polysomnography (PSG). PSG is an essential method for diagnosis in patients who have symptoms and clear comorbidities, such as chronic obstructive pulmonary disease or ischaemic stroke, as well as those with a clinical record suggestive of a sleep disorder. OSAS has become a significant public health problem, but investment in treatments for OSAS remain inadequate.^1^

This method has been proven to be accurate with a low failure rate because the study is attended by technical staff; PSG, however, is considered relatively expensive and technically complex. The AASM classified sleep study devices.^2^, ^3^ While portable monitoring devices are less reliable overall than attended devices, they may be used at the patient’s home. While no optimal portable monitoring device exists, a device must at least supply sufficiently certain diagnostic measurements and be adaptable for use at home by inexperienced patients. Portable monitoring (PM) has been utilized as an alternative diagnostic test for obstructive sleep apnoea based in part on the premise that it is less expensive and quicker to deploy compared to in-laboratory PSG. However, there is a paucity of evidence that shows PM is equivalent to PSG in regards to diagnosis, treatment, and outcomes. In most reported literatures, it was stated that PM can be as accurate as PSG for diagnosis in selected populations candidate for PM.^4^-^12^

In this study, we compared a portable monitoring device with PSG for the measurement of parameters related to diagnosis of OSAS. The device used a novel computer algorithm based on a combination of oxygen saturation and photoplethysmography via pulse wave analysis to detect both respiratory and non-respiratory sleep disorders.

## METHODS

### Study Group

This study consisted of 155 adult OSAS patients (30 women and 125 men, mean age, 52.18 ± 12.31 years) who visited the Hendek Government Hospital Sleep Laboratory between February 2012 and January 2013.

### OSAS Diagnosis

A detailed account of the methodology is available in the text supplement. The study protocol was approved by the institutional research ethics committee of the University of Sakarya, Turkey, and participants provided written informed consent. The study was designed to meet ASDA 2007 guidelines for reports of diagnostic accuracy.^2^

### Inclusion & Exclusion Criteria

Individuals showing infiltration in their lung radiograph or other systemic diseases were excluded from the study.

### Study Design

Before taking the sleep test, all OSAS patients completed a questionnaire regarding sleep problems to determine symptoms which may have developed for their sleep disorder. Using this form, basic OSAS symptoms including snoring, witnessed apnoea, and day time sleepiness were evaluated. To objectively evaluate excessive daytime sleepiness, the Epworth Sleepiness Scale (ESS) was used.^13^ An ear–nose–throat examination was performed to all patients.

The patients were examined by a trained sleep technician at the Sleep Laboratory who took anthropometric measurements and attached sleep recording devices to the patients. The compact screening device Model Somnocheck Micro (Type 4 device), 1358-1362-1367 (SM) (Weinmann Medical Technology, Hamburg, Germany) was attached to the patient’s wrist. A combination of photoplethysmography-derived pulse wave analysis and respiratory flow signals may enable differentiation between obstructive and central apnoea and provide information regarding the extent of sleep fragmentation. In detail, respiratory effort was derived by analyzing fluctuations of the PWA signal caused by intrathoracic pressure changes during spontaneous breathing cycles.^4^, ^5^, ^14^, ^15^

After evaluating all of the patients’ reports in the study, appointments were made for the patients for an overnight PSG test at the sleep laboratory. On the day of the laboratory stay, the patients were advised not to sleep during the day, not to consume any caffeinated beverages or food, and not to use alcohol and drugs such as antihistamines, antidepressants, or hypnotics which would affect their sleep patterns.

### PSG model

Somte PSG (Type 1 device), Ser-No: 3127 CAB2-06; Compumedics, Melbourne, Australia. A single experienced chest clinician performed manual scoring of all PSGs according to internationally defined criteria. Detailed descriptions of the Somte PSG device and scoring criteria are provided in the online supplement.

### OSAS-related Definitions

### Apnoea

A complete lack of air flow through the mouth and nose for ≥10 s.

Hypopnea: Less than or equal to 50% air flow for ≥10 s, along with a 3% decrease in oxygen saturation or development of arousal.

### Apnoea–Hypopnea Index (AHI)

The ratio obtained by dividing the total duration of apnoea and hypopnea observed during sleep by the total duration of sleep.

### OSAS Severity

Determined on the basis of AHI. Normal: AHI < 5/h. Mild sleep apnoea: AHI between 5 and 15/h. Moderate sleep apnoea: AHI between 16 and 30/h. Severe sleep apnoea: AHI > 30/h.

### Desaturation index

Number of oxygen desaturations within the artifact-free evaluation time of the pulsoximetry signal.

**MeanO_2_;** Average oxygen saturation within the artifact-free evaluation time of the pulsoximetry signal.

**MinO_2_;** Minimum oxygen saturation within the artifact-free evaluation time of the pulsoximetry signal

### Statistical Analysis

Two paired sample t-tests were used to compare the AHI, meanO_2_, minO_2_, and desaturation index (DI) values between the SM and PSG methods. Continuous data were presented as the mean ± standard deviations. A marginal homogeneity test was used to compare the grouped AHI, grouped meanO_2_, grouped minO_2_, and grouped DI between the SM and PSG methods. Kendall tau-c (τc) coefficients were used for the determination of the concordances between the SM and PSG methods. In addition to this, correlation among AHI, meanO_2_, minO_2_, and desaturation index (DI) values were investigated with Bland&Altman plots method which was suggested by Bland and Altman. By using SM method AHI, meanO_2_, minO_2_, and desaturation index(DI) values were calculated. PSG was used in these results. According to these results, correlation limits (mean ± 1.96 SD) were investigated.^16^, ^17^ Commercial software (IBM SPSS Statistics 23; SPSS Inc. and IBM Corp., Armonk, NY; Med Calc 16.2.1; Med Calc Software BVBA, Ostend, Belgium) was used to perform the analyses. A p-value < 0.05 was considered to indicate statistical significance.

Informed consent was obtained from all subjects, and the study was approved by the Sakarya University Faculty of Medicine Ethics Committee.

## RESULTS

There were 125 male and 30 female (19.4%) patients. The demographic and clinical features of the patients are summarized in Table-I. When questioned about OSAS major symptoms, 81.9% (127/155) of patients had snoring, and in 54.8% (85/155), this snoring was determined to be habitual. Daytime sleepiness was found in 51.6%(80/155) and witnessed apnea was found in 37.4%(58/155). The demographic and clinical features of the patients are summarizedin Table-I.

**Table-I:** General patient characteristics.

Age	52±12
Sex Male	125 (80.6%)
Female	30 (19.4%)
Body Mass Index	32.79 ± 5.17
Epworth Sleepiness Scale	14.6 ± 4.2
Habitual snoring	54.8%(84/155)
Daytime sleepiness	51.6%(80/155)
Witnessed apnoea	37.4%(58/155)
Smoking	
Current smoker	50 (32.5%)
Non-smoker	78 (50.6%)
Ex-smoker	26 (16.9%)
Glucose(mg/dl)	108.02 ± 29.7
Total Cholesterol(mg/dl)	204.95 ± 53.45
Triglycerides(mg/dl)	187.5 ± 73.39
Low DensityLipoprorein(mg/dl)	124.54 ± 43.29
HighDensityLipoprotein(mg/dl)	42.38 ± 10.73
C-Reactive Protein(mg/L))	4.84 ± 4.92
Hemoglobin (g/dl)	14.31 ± 1.85
Hemotocrit(%)	43.45 ± 5.24

Data shown as n (%) and mean ± standard deviation.

**Fig.1:**
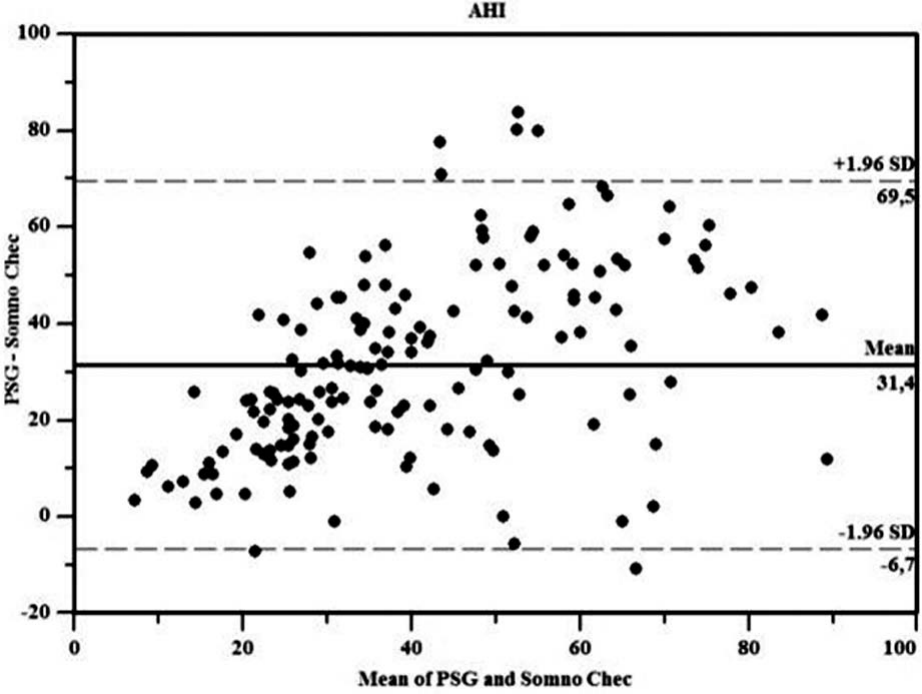
Bland & Altman plots for AHI.

We found differences between meanO_2_ and DI, found no differences between AHI and minO_2_ upon comparison of SM and PSG measurement methods. MinO_2_ level was the same in both methods because there was no timed index value

AHI, meanO_2_, minO_2_, and desaturation indeks (DI) values’ scatter plot (Bland & Altman plots) drawn between the differences and averages (These values were obtained by the methods of SM and PSG) is between ±1.96 SD. Since it is between±1.96 SD, correlations was seen between two methods.

## DISCUSSION

In this study, we compared the SM portable monitoring device with PSG for measurement of parameters related to the diagnosis of OSAS. We found differences in the meanO_2_ and DI, but found no difference in the AHI and minO_2_ between the two measurement methods. Differences between the methods were not desired. However, the relationship between the methods, showing differences in the measured values, supported our hypothesis. The high level of O_2_ saturation in the SM measurements was due to the fact that the patients spent a greater number of hours in the awakened state. AHI, DI, and meanO_2_ levels in the SM group were low for the same reason. MinO_2_ levels were the same in both methods because there was no timed index value. Therefore, even when a patient slept for a short time, the results were not affected.

**Fig.2:**
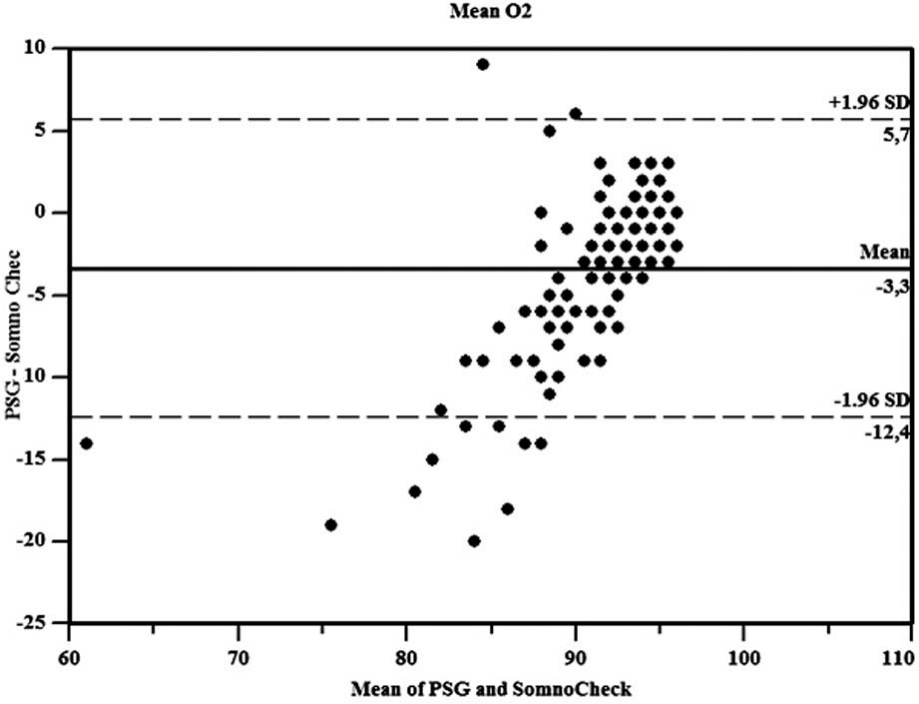
Bland & Altman plots of mean O_2_.

**Table-II:** Comparison and concordance analysis results of the 2 methods for the grouped data.

		SM	PSG	p-values	Concordance	

					Tc	p-values
AHI	AHI < 5	7 (4,5)	-	0.056	0.203	<0.001
	5 < AHI < 15	45 (24,5)	4 (2,6)			
	15 < AHI <30	61 (39,4)	14 (9)			
	30 < AHI	49 (31,6)	137 (88,4)			
MeanO_2_	95 < O_2_	56 (36,1)	20 (12,9)	<0.001	0.329	<0.001
	93 < O_2_ <95	56 (36.1)	41 (26.5)			
	91 < O_2_ <93	26 (16.8)	33 (21.3)			
	89 < O_2_ < 91	9 (5.8)	21 (13.5)			
	O_2_< 89	8 (5.2)	40 (25.8)			
MinO_2_	80 < O_2_ < 90	43 (27.7)	56 (36.1)	0.117	0.501	<0.001
	70 < O_2_ < 80	50 (32.3)	45 (29)			
	60 < O_2_ < 70	37 (23.9)	27 (17.4)			
	O_2_ < 60	25 (16.1)	27 (17.4)			
DI	O_2_ DI < 5	13 (8.4)	10 (6.5)	0.027	0.242	<0.001
	5 < O_2_ DI < 15	27 (17.4)	22 (14.2)			
	15 < O_2_ DI < 30	39 (25.2)	27 (17.4)			
	30 < O_2_ DI	76 (49.0)	96 (61.9)			

Data shown as n (%). Statistically significant p values are shown in bold. a: p values of the comparison between 2 methods (marginal homogeneity test). b: p values of the concordance of the 2 methods (Kendall tau-c). AHI: apnoea hypopnea index, DI: desaturation index, MeanO_2_: Average oxgen saturation MinO_2_: minimum oxygen saturation, O_2_: Oxygen, SM:somnocheck micro, PSG: polysomnography

**Fig.3:**
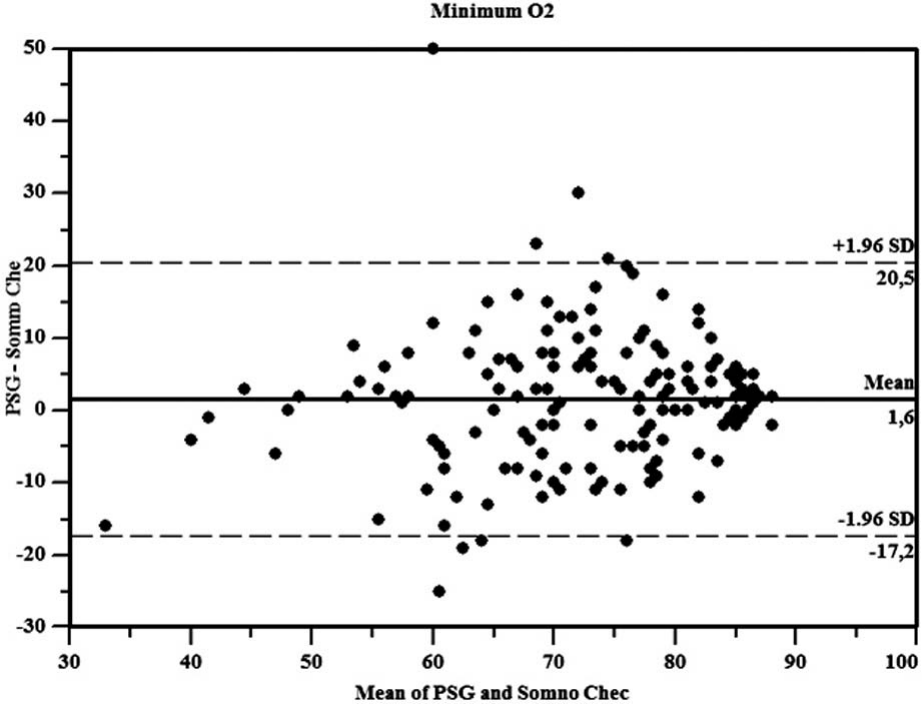
Bland& Altman plots for minimum O_2_.

Nocturnal oximetry may be used as a screening method because it may demonstrate the presence of apnoea or hypopnea, but does not distinguish between central or devices record in the sleep state, and both the AHI and arousals are obstructive disorders. Furthermore, it does not detect events without desaturation. Therefore, the use of nocturnal oximetry as a single diagnostic method is not recommended. None of these four type are commonly underestimated. Various surrogate measures of arousals such as actigraphy have been shown to improve the arousal index and possibly the agreement between OSAS diagnosis and PSG.^5^, ^6^ Any improvement may only be slight and not clinically important, as reported by Masa et al.^7^ in case of a type 3 device. In recent years, such devices have been validated for PSG at different sensitivities and specificities, depending on the device or AHI cut off value.^8^, ^9^

**Fig.4:**
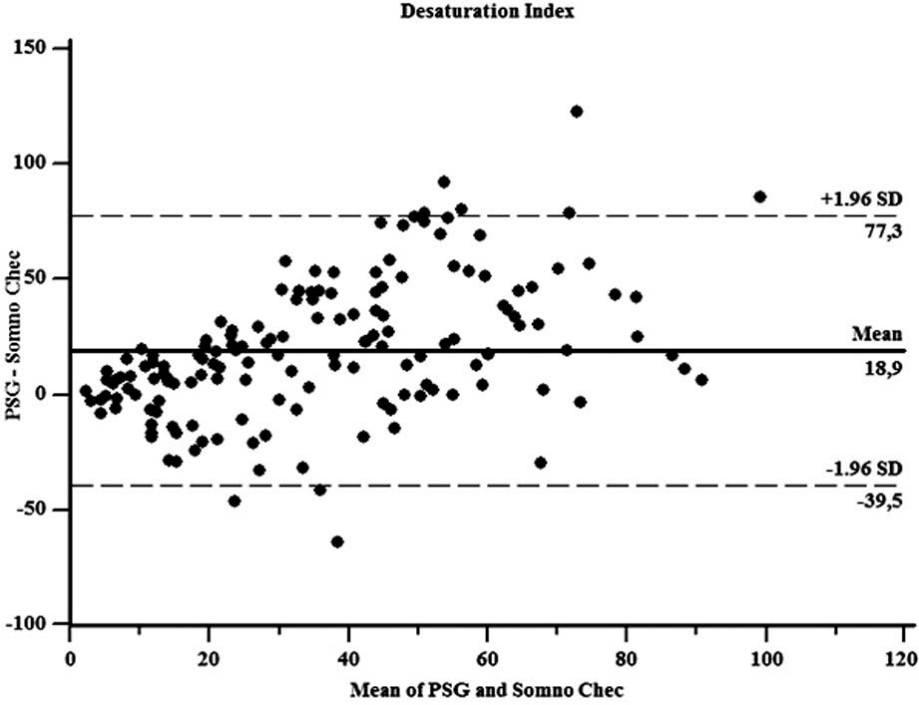
Bland& Altman plots for DI.

The results of these studies have shown that single-channel nasal airflow pressure can be used as a definite alternative diagnostic tool in this population, either at home or in a sleep clinic.

OSAS is more prevalent than asthma, chronic obstructive pulmonary disease (COPD), or diabetes. OSAS must be conducted at different health levels, because OSAS is already a widespread concern for public wellness. Cost analyses are complicated by the variability of the type 4 devices. This analysis has demonstrated cost savings from the use of oximetry, but the considerable loss of diagnostic certainty did not eventually allow for a cost-effective approach.^10^

A study was conducted on the use of a portable monitoring device (Somnocheck Micro) for the research and diagnosis of obstructive sleep apnoea: comparison with polysomnography. The apnoea/hypopnoea index (AHI) acquired by manual SC analysis correlated closely with that obtained by PSG (r = 0.98).^18^

## CONCLUSIONS

The results of our study have shown that the SM portable device can be used as an alternative diagnostic tool in this population either at home or in sleep clinic.
